# On the potential of virtual reality for locomotion rehabilitation

**DOI:** 10.1080/07853890.2021.1896637

**Published:** 2021-09-28

**Authors:** Alexandre Gordo, Inês dos Santos Silva, Hugo Nicolau, Daniel Simões Lopes

**Affiliations:** INESC-ID, Instituto Superior Técnico, Universidade de Lisboa, Lisbon, Portugal

## Abstract

**Introduction:**

In recent years, we have witnessed a growing number of people needing locomotion rehabilitation (e.g. stroke). The inability to walk has tremendous effects on the individuals' wellbeing and quality of life, making locomotion rehabilitation a vital component of physiotherapy. Virtual reality (VR), a term used to describe a technological system that creates a simulated world or environment, is a promising technology that has proven significant benefits in rehabilitation [1]. Our research goal is to help physiotherapists include VR in locomotion rehabilitations. In this work, we built an immersive VR system, Locomotiver, where users believe they are present in another specific environment. Locomotiver aim to support locomotion rehabilitation and fit physiotherapists' practices and patients' abilities, which included customisable exercises for lower limb recovery. These exercises mapped real ones from traditional interventions, also provide a very engaging and motivating experience for the users.

**Materials and Methods:**

Locomotiver consists in a VR environment that can be used by therapists and patients, simultaneously. The patient experiences a 360° immersive environment using an HTC Vive headset and four trackers. Kondo et al. [2] inspired us to the minimalistic designed representation of the virtual body, representing the patients’ head, hands, and feet in the virtual world. Furthermore, we did field research, where we observed the therapists working with several neurological and musculoskeletal patients and perform a variety of exercises related to their locomotion therapies. Also, based on formative series studies, we created three exercises (“Walking Forward”, “Barriers” and “Zigzag”), customisable to fit the patients’ abilities and session goals. Also, the physiotherapist can observe the patient’s 3rd person or 1st person perspective, along a graphical user interface to control the rehabilitation session. Corrective feedback is given during the performance of exercises while therapists have access to a set of measures, which can then be leveraged to build personalised therapy plans. Also, the physiotherapist can observe the patient’s 3rd person or 1st person perspective, along a graphical user interface to control the rehabilitation session. We conducted a usability study with nine physiotherapists using a think-aloud protocol, semi-structured interviews, and adoption questionnaires, aiming to understand Locomotiver’s potential to be deployed in the field. We did a thematic analysis of the interviews and the feedback collected during the experiments. The Ethics Committee of Egas Moniz approved this study (process number 657). All participants signed consent forms.

**Results:**

Physiotherapists agreed that Locomotiver would be an innovative solution to their interventions and to increase patients’ engagement. They agreed that Locomotiver is more proper for patients with musculoskeletal disorders than for neurological patients. Therapists also stated that exercises need to allow further customisation and collect additional performance data. Participants praised both the instruments and prototype, namely how fast and easy they were to set up, compared to other conventional systems. Overall, professionals show high interest in adopting Locomotiver, pointing benefits such as optimisation of their methods, ease of customisation, and improved diagnosis.

**Discussion and conclusions:**

We presented Locomotiver, an immersive VR system for locomotion rehabilitation that includes three customisable exercises. We verified that Locomotiver an engaging and motivating experience for the users. We concluded that professional therapists would be interested in adopting Locomotiver as a rehabilitation tool. This research contributes to highlight key challenges and opportunities when introducing immersive VR technologies in clinical rehabilitation practices. We believe that this research contributes to establish this a baseline to develop and introduce immersive VR technology that significantly increases the motivation of patients, optimises intervention processes and improves the overall locomotion rehabilitation. As future work, we propose that physiotherapists use Locomotiver with patients for a certain period, in a real-life scenario.
